# Comparative Skull Analysis Suggests Species-Specific Captivity-Related Malformation in Lions (*Panthera leo*)

**DOI:** 10.1371/journal.pone.0094527

**Published:** 2014-04-09

**Authors:** Joseph Saragusty, Anat Shavit-Meyrav, Nobuyuki Yamaguchi, Rona Nadler, Tali Bdolah-Abram, Laura Gibeon, Thomas B. Hildebrandt, Merav H. Shamir

**Affiliations:** 1 Department of Reproduction Management, Leibniz Institute for Zoo and Wildlife Research, Berlin, Germany; 2 Koret School of Veterinary Medicine, The Robert H. Smith Faculty of Agriculture, Food and Environment, The Hebrew University of Jerusalem, Rehovot, Israel; 3 Wildlife Conservation Research Unit, Department of Zoology, University of Oxford, Tubney, Abingdon, United Kingdom; 4 Zoological Center Tel Aviv-Ramat Gan, Ramat Gan, Israel; 5 NYC Veterinary Specialists, New York, New York, United States of America; University of Illinois at Urbana-Champaign, United States of America

## Abstract

Lion (*Panthera leo*) populations have dramatically decreased worldwide with a surviving population estimated at 32,000 across the African savannah. Lions have been kept in captivity for centuries and, although they reproduce well, high rates of stillbirths as well as morbidity and mortality of neonate and young lions are reported. Many of these cases are associated with bone malformations, including foramen magnum (FM) stenosis and thickened tentorium cerebelli. The precise causes of these malformations and whether they are unique to captive lions remain unclear. To test whether captivity is associated with FM stenosis, we evaluated 575 lion skulls of wild (*N* = 512) and captive (*N* = 63) origin. Tiger skulls (*N* = 276; 56 captive, 220 wild) were measured for comparison. While no differences were found between males and females or between subadults and adults in FM height (FMH), FMH of captive lions (17.36±3.20 mm) was significantly smaller and with greater variability when compared to that in wild lions (19.77±2.11 mm). There was no difference between wild (18.47±1.26 mm) and captive (18.56±1.64 mm) tigers in FMH. Birth origin (wild vs. captive) as a factor for FMH remained significant in lions even after controlling for age and sex. Whereas only 20/473 wild lions (4.2%) had FMH equal to or smaller than the 5^th^ percentile of the wild population (16.60 mm), this was evident in 40.4% (23/57) of captive lion skulls. Similar comparison for tigers found no differences between the captive and wild populations. Lions with FMH equal to or smaller than the 5^th^ percentile had wider skulls with smaller cranial volume. Cranial volume remained smaller in both male and female captive lions when controlled for skull size. These findings suggest species- and captivity-related predisposition for the pathology in lions.

## Introduction

The lion (*Panthera leo*) once ranged across large parts of Africa, Europe, the Middle East and Asia. It has since become increasingly endangered in the wild with an estimated population of 32,000–35,000 free ranging lions left in Africa (*P. leo leo*) [1] and approximately 350 in Asia (*P. leo persica*) [2]. Most lions live in savannah habitats across sub-Saharan Africa and a sizable population is kept in zoological gardens all over the world [3]. Lions are known to reproduce well in captivity but high incidence of morbidity and mortality has been reported in young captive lions [4]. For example, of the 126 captive born lions with known outcome listed in the 2002 North American studbook, 51 (40.5%) died before the age of two years (age of sexual maturity). Of these deaths, eight (15.7%) resulted from infanticide; five (9.8%) were registered as stillbirth; 37 (74.5%) were of unknown cause, and one animal was euthanized for unknown reason [5]. These numbers are similar to those reported by Haas et al. [6] and Clubb and Mason [7]. A sizable number of these cases have been attributed to bone malformations, primarily of the skull, that include thickening of the tentorium cerebelli and occipital bone, and narrowing of the foramen magnum (FM) [8]–[16]. These changes apply pressure on the nervous tissue at the caudal fossa, causing severe, and potentially fatal, neurologic abnormalities [8], [10], [11]. A decrease in FM height has been documented in captive lion skulls dating as far back as the 15^th^ century [9]. Skull abnormalities have been reported over the years in lions from zoological gardens in Europe [17], South Africa [11], Australia [8], the United States [10], [18], and Asia [19]. Whether these malformations also occur in wild lions and whether they are caused by environmental or genetic factors, or a combination of both, has yet to be determined. It is important to note that such morphological abnormalities have not been reported to date from wild lion populations and have only been rarely described in non-lion large felids in captivity (cheetah, *Acinonyx jubatus* [20]; tiger, *P. tigris* [21]).

The present study is the first to investigate FM malformation in a large number of lion skulls from various geographic regions in the wild, comparing them to skulls of lions in captivity and to tiger skulls of both captive and wild origin, the tiger representing a similar-sized obligatory carnivore. By comparing measurements of the skulls between wild and captive lions and tigers, our objective was to test the hypothesis that lions under captive conditions frequently present abnormal bone growth of the skull, resulting in increased risk for FM stenosis and thus being predisposed to neurologic disease.

## Materials and Methods

### Skull measurements

Lion and tiger skulls kept in natural history collections in Africa, Europe, and North America (see Acknowledgements for details) were examined. All specimens were accessed by permission of the respective museums and were either studied at the museum's premises or were loaned for data acquisition. Based on museum labels, specimens were categorized for sex, geographic origin, and whether of wild or captive origin at time of death. Age categories were often missing. In such cases, a skull was classified as juvenile if the cemento-enamel junction of any permanent canine was not clearly visible above its alveolus. If these junctions were visible and yet the basioccipital-basisphenoid suture and/or the frontal suture were still open, a skull was classified as subadult. If these sutures were closed, a skull was classified as adult [22]. Only adults and subadults were used for analysis, owing to small sample sizes for juveniles. Information on the clinical history of the specimens or their background (date of birth or death, if they were captive-born or wild-caught, who were their parents, and clinical history) was not available.

**Figure 1:**
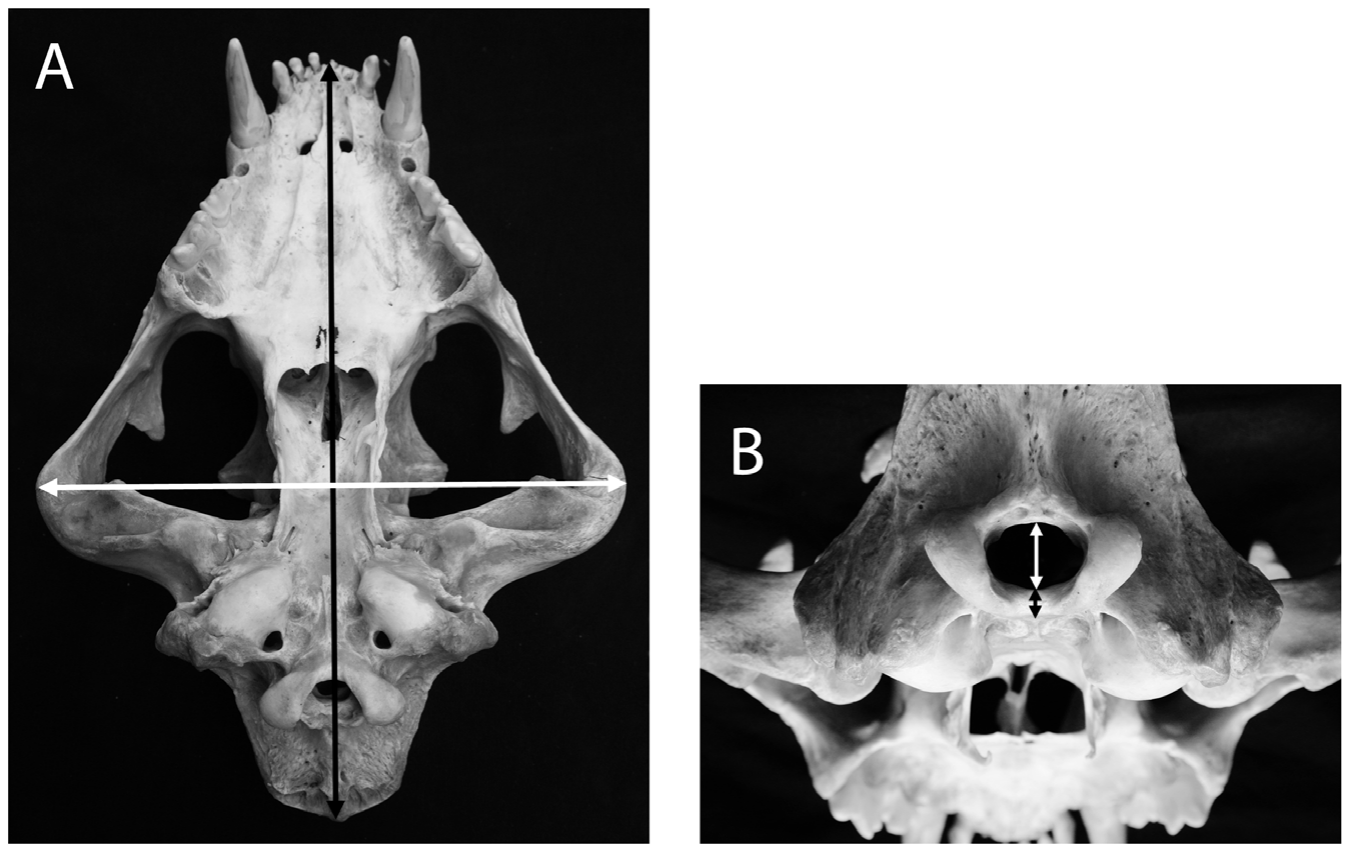
A - The distance between the posthion and the inion - skull length (SL) - black arrow. The distance between the left and right zygomatic arches - skull width (SW) - white arrow. **B** - The distance between the top of the basilar part of the occipital bone and the bottom of the occipital bone - foramen magnum height (FMH) – white arrow. The distance between the bottom and the top of the basilar part of the occipital bone at its most caudal aspect - basilar thickness (BT) – black arrow.

Four measurements were taken from the skulls (Figure 1), using metal caliper: (1) Skull's length (SL): Distance between the prothion and the inion. (2) Skull's width (SW): Distance between the left and right zygomatic arches at the widest point. (3) FM height (FMH): Distance from the top of the basilar part of the occipital bone to the bottom of the occipital bone measured at the midline. (4) Basilar thickness (BT): Distance between the bottom and top of the basilar part of the occipital bone at its most caudal aspect. A fifth measurement, cranial volume (SV), was taken as follows: The cranial cavity was cleaned and filled with glass balls of c. 4 mm diameter, which were then either weighed using an electronic balance to the nearest 1 g, or measured using a plastic cylinder to the nearest 1 cm^3^. Weights were converted into volumes using the equation: *Volume* = *0.633 × Weight + 0.939* based on linear regression coefficient (*R^2^* = 1.000, *DF* = 1, *F* = 24172.7, *P*<0.001).

### Statistical analysis

Statistical analysis was performed using PASW Statistics 18 (IBM Inc., Chicago, IL). Wherever clear differences have been known, or were detected, between animal categories (e.g. age or sex classes), each animal category was analyzed separately. Otherwise, data were pooled for analysis. Levene's Test for Homogeneity of Variances and the Bartlett's test were used to compare the variance between different groups in continuous variables. Pearson's correlation coefficient was calculated in order to check the correlation between two quantitative variables. *T*-test and Mann-Whitney *U*-test were used to compare between two groups of quantitative variables. Fisher's exact test was used to test the association between two categorical variables. To assess simultaneously the effect of several variables on a quantitative dependent variable, Analysis of Covariance (ANCOVA) model was applied. Quantitative data are presented as arithmetic means, standard deviations (SD) and coefficient of variation (CV = SD/Mean).

**Figure 2:**
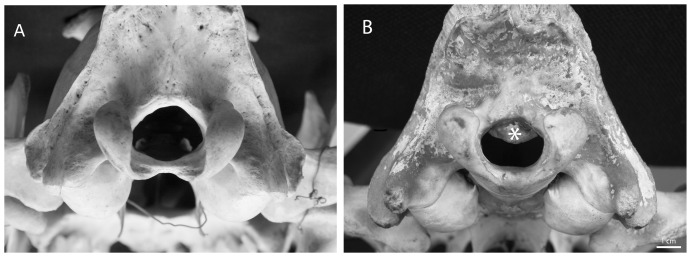
Two captive lion skull specimens. **A**– A skull of an adult lion with a normal opening of the foramen magnum. **B** - Abnormal bone growth protruding down from the roof of the foramen magnum (asterisk) in a young adult lion. (Figure 2A courtesy of Mr. Tom Kouris).

Following our hypothesis that skulls with FMH larger than the 5^th^ percentile would probably have a normal FM opening (Figure 2A), the 5^th^ percentile of the studied skulls was chosen as the divide between skulls with a potentially normal opening and those with FM stenosis. The 5^th^ percentile point is commonly used as a cutoff in clinical studies [23]–[26]. Since the wild populations presented normal distribution in FMH, the 5^th^ percentile of FMH for these populations, which is equal to any measurement of more than 1.23 SD below the mean for lions and 1.44 SD below the mean for tigers, were determined to be 16.60 mm for lions and 16.54 mm for tigers. As a second approach, we presently have actual measurements of the FMH in six lions confirmed to have FM stenosis-related neurological dysfunction (all captive; their skulls are not included in this study). The maximal FMH amongst these lions is 14.06 mm (median: 12.56 mm, range: 11.10–14.06 mm). Comparison was also conducted between captive and wild lions based on this value as the cutoff between skulls with potentially normal opening and those with FM stenosis.

All tests applied were two-tailed and, where relevant, exact. A *P*-value <0.05 was considered statistically significant for all tests.

## Results

**Figure 3:**
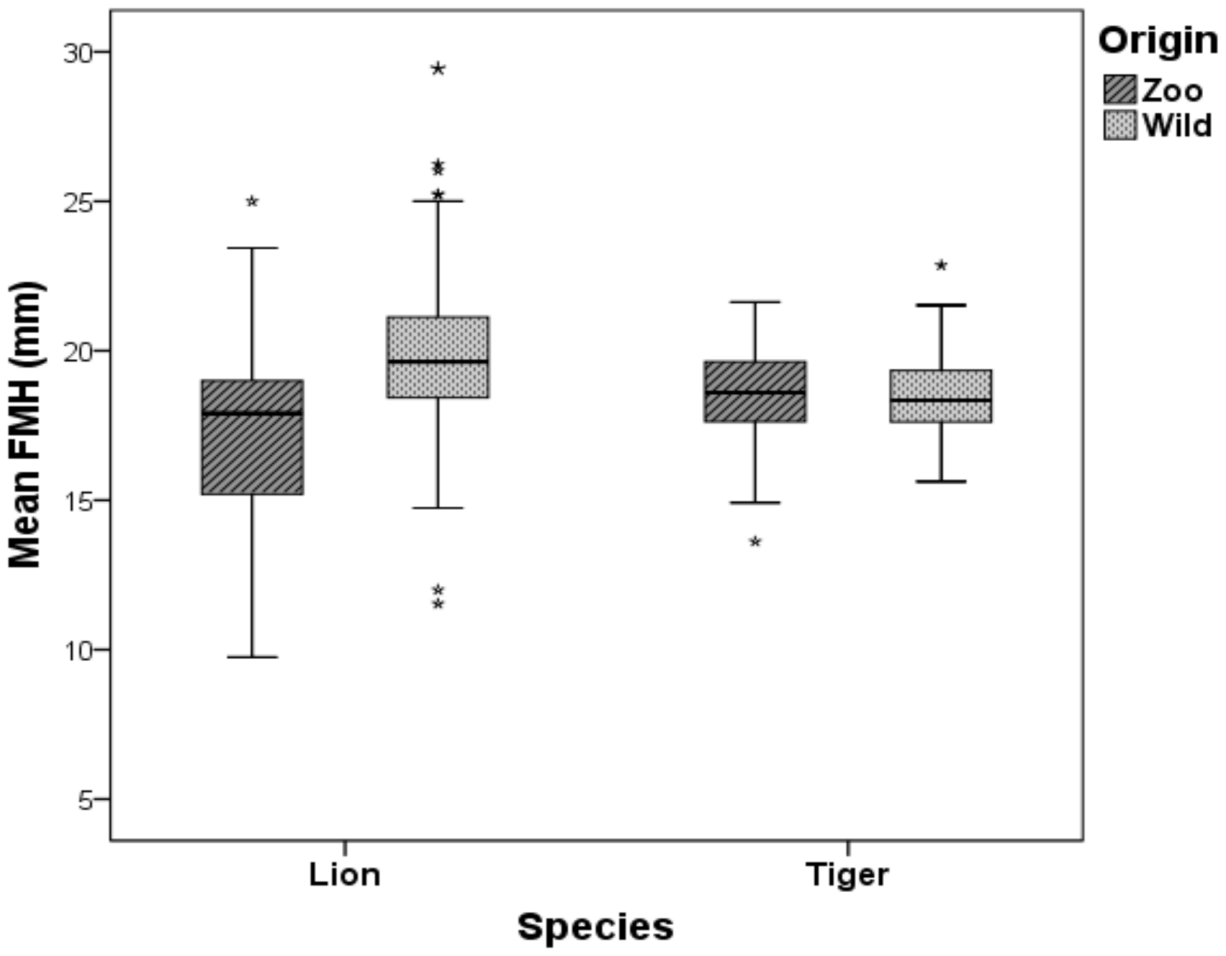
The mean foramen magnum height (FMH) in captive lions is significantly lower than that of lion skulls from wild populations (17.36±3.20 and 19.77±2.11 respectively; *T*-test: t = 5.543, *DF* = 61.969, *P*<0.001). No statistically significant difference was noted between the captive and wild tiger populations. Asterisks represent outliers.

A total of 851 skulls of known captive or wild status at death were included in this study. These comprised 575 lion skulls and 276 tiger skulls. The lion specimens consisted of 63 skulls of captive animals and 512 of wild ones whilst for tigers there were 56 and 220 skulls of captive and wild origin, respectively. There was no statistically significant difference in mean FMH between males and females, or between adults and subadults, in either species (Table 1). There was also no statistically significant correlation between mean FMH and skull size (represented by SL) in either males or females in both species (Table 2). Therefore, FMH data of both sexes and age classes were combined for further analysis. Mean FMH in captive lions was significantly smaller than that of wild lions (17.36±3.20 mm and 19.77±2.11 mm, respectively; *T*-test: t = 7.653, *DF* = 528, *P*<0.001) (Figure 3; Table 1). In contrast, no statistically significant difference was found in FMH between captive and wild tigers (Table 1). The lions' origin (wild or captive), controlled for age and sex, remained significant in explaining FMH differences (ANCOVA: *DF* = 1, *F* = 50.486, *P*<0.001).

**Table 1:** Mean FMH (mm) in lions and tigers.

Lions		Tigers	
*Wild*	*Captive*	*Wild*	*Captive*
19.77±2.11^a*^	17.36±3.20^a*^	18.47±1.26^b^	18.56±1.64^b^
*Subadult*	*Adult*	*Subadult*	*Adult*
19.57±2.65^c^	19.54±2.39^c^	18.53±1.00^d^	18.59±1.35^d^
*All males*	*All females*	*All males*	*All females*
19.41±2.53^e^	19.60±2.12^e^	18.58±1.36^f^	18.32±1.42^f^
*All adult males*	*All adult females*	*All adult males*	*All adult females*
19.46±2.63^g^	19.58±2.17^g^	18.64±1.39^h^	18.54±1.37^h^
*Adult wild males*	*Adult wild females*	*Adult wild male*	*Adult wild females*
19.84±2.27^i^	19.82±2.03^i^	18.77±1.19^j^	18.44±1.27^j^
*Adult captive males*	*Adult captive females*	*Adult captive males*	*Adult captive females*
17.15±3.41^k^	17.87±2.42^k^	18.28±1.81^l^	18.75±1.58^l^

Parameters with superscript asterisk were statistically different.

Values are presented as mean ± SD.

All comparisons were done using *T*-test:

a. t = 5.543, *DF* = 61.969, *P*<0.001

b. t = 0.372, *DF* = 73.150, *P* = 0.711

c. t = 0.090, *DF* = 462, *P* = 0.928

d. t = 0.189, *DF* = 214, *P* = 0.850

e. t = 0.887, *DF* = 482, *P* = 0.376

f. t = 1.316, *DF* = 208, *P* = 0.190

g. t = 0.495, *DF* = 368, *P* = 0.621

h. t = 0.436, *DF* = 164, *P* = 0.663

i. t = 0.087, *DF* = 319, *P* = 0.931

j. t = 1.400, *DF* = 116, *P* = 0.164

k. t = 0.827, *DF* = 47, *P* = 0.412

l. t = 0.945, *DF* = 46, *P* = 0.349

**Table 2:** Pearson Correlations between FMH and SL and between SL and SW in lions and tigers.

			*N* FMH	*N* SL	*N* SW	r	*P*
**Lions**	***Correlations between FMH and SL***						
	**Females**	**Adults & subadults**	187	130		0.054	0.551
		**Adults**	173	121		0.020	0.837
	**Males**	**Adults & subadults**	234	169		0.033	0.685
		**Adults**	197	148		0.056	0.518
	***Correlations between SL and SW***						
	**Females**	**Adults & Subadults**		130	192	0.525	<0.001
		**Adults**		121	178	0.483	<0.001
	**Males**	**Adults & Subadults**		169	247	0.207	0.002
		**Adults**		148	208	0.528	<0.001
**Tigers**	***Correlations between FMH and SL***						
	**Females**	**Adults & subadults**	72	66		0.157	0.231
		**Adults**	68	62		0.150	0.270
	**Males**	**Adults & subadults**	108	94		0.056	0.601
		**Adults**	98	83		0.032	0.774
	***Correlations between SL and SW***						
	**Females**	**Adults & subadults**		66	77	0.832	<0.001
		**Adults**		62	73	0.816	<0.001
	**Males**	**Adults & subadults**		94	112	0.824	<0.001
		**Adults**		83	101	0.805	<0.001

FMH  =  foramen magnum height, SL  =  skull length, SW  =  skull width.

Lions showed greater heterogeneity in FMH (SD = 2.37 and CV = 0.121) when compared to tigers (SD = 1.35 and CV = 0.073) (Levene's test: *F* = 46.033, *P*<0.001). Captive lions had significantly greater heterogeneity in FMH than wild lions (SD = 3.20 and CV = 0.184, and SD = 2.11 and CV = 0.106, respectively; Levene's test: *F* = 22.28, *P*<0.001). Differences remained highly significant when only skulls from adult animals were compared (data not shown).

In lions, 23 out of the 57 skulls of captive animals examined (40.4%) were found to have FMH equal to or smaller than the 5^th^ percentile of the wild population (16.60 mm). In comparison, only 4.2% (20/473) of skulls from the wild lion population fell under this category. The difference between these proportions was highly significant (Fisher's exact test: *P*<0.00000001). Differences remained highly significant even when only adult skulls were compared (Fisher's exact test: *P*<0.00000001). When the same comparison was conducted using 14.06 mm (maximal FMH amongst six confirmed cases of FM stenosis with related neurological illness in lions) as the cutoff value, 2/473 (0.4%) of lion skulls from the wild had FMH smaller than this value while there were 9/57 (15.8%) skulls of lions from captivity in this category. The difference between these two proportions was still highly significant (Fisher's exact test: *P* = 0.000000049). Differences remained highly significant even when only adult skulls were compared (Fisher's exact test: *P*<0.0000081). Of the 43 lion skulls with constricted FM, some showed bone growth protruding ventrally from the roof of the FM (Figure 2B). Among tigers, there were 5/55 (9.1%) skulls from captivity and 10/194 (5.2%) skulls from the wild with FMH equal to, or smaller than, the 5^th^ percentile of the wild population in this species (16.54 mm). There was no statistically significant difference in the proportions between these two groups (Fisher's exact test: *P* = 0.333).

To check if these differences were due to skulls of lions with small FMH being smaller in general, we also compared their SV, SL, SW and BT values to skulls with FMH larger than 16.60 mm (Table 3). Interestingly, SW was significantly larger and SV was significantly smaller in the skulls with narrow FM. However, there appears to be a positive correlation between SL and SV [22] potentially influencing the results. We therefore compared SV/SL ratio between skulls whose FMH are either smaller or larger than the 5^th^ percentile. Lions with FMH smaller than the 5^th^ percentile had significantly smaller SV/SL ratio in both males (Mann-Whitney *U* test: *N* = 12 & 147, *U* = 440, *P* = 0.003) and females (Mann-Whitney *U* test: *N* = 5 & 116, *U* = 74, *P* = 0.003). Although male lion skulls of captive origins had significantly smaller SV/SL ratio than did those from the wild (Mann-Whitney U test: *N* = 15 & 126, *U* = 565, *P* = 0.01) there were no significant differences in female lions or in male or female tigers. This difference between male lions skulls no longer existed when only skulls with FMH larger than the 5^th^ percentile were compared between captive and wild animals (Mann-Whitney U test: *N* = 9 & 118, *U* = 419, *P* = 0.300).

**Table 3:** Measurements of lion skulls with FM stenosis (FMH< = 16.60 mm) and those with larger FM opening, comparing males and females separately.

	Males		Females	
	*FMH< = 16.60*	*FMH>16.60*	*FMH< = 16.60*	*FMH>16.60*
Cranial volume	250.97±20.29^a*^	263.24±17.72^a*^	204.20±15.34^b*^	228.67±14.75^b*^
BT thickness	9.63±2.55^c^	8.86±1.61^c^	7.99±0.90^d^	6.99±1.48^d^
Skull width	245.85±22.66^e*^	229.92±24.48^e*^	205.12±15.21^f*^	195.16±14.38^f*^
Skull length	365.03±24.97^g^	355.09±21.09^g^	291.16±10.66^h^	295.95±14.02^h^

Significantly different values in the same raw and within the same sex are marked with a superscript asterisk.

FMH  =  foramen magnum height, BT  =  basilar thickness

All comparisons were done using *T*-test:

a t = 2.284, *DF* = 159, *P* = 0.024

b t = 3.630, *DF* = 120, *P*<0.001

c t = 1.339, *DF* = 72, *P* = 0.185

d t = 1.468, *DF* = 61, *P* = 0.147

e t = 3.283, *DF* = 265, *P* = 0.001

f t = 2.229, *DF* = 205, *P* = 0.027

g t = 1.826, *DF* = 192, *P* = 0.069

h t = 0.826, *DF* = 145, *P* = 0.410

## Discussion

Studies on captive lions that have died following neurologic abnormalities revealed severe reductions in the FMH, accompanied by thickening of the tentorium cerebelli and the basal part of the occipital bone [8], [10], [11], [16], [17], [19]. Results of this study suggest that FMH is smaller in skulls of captive lions than in skulls of wild lions but not in tigers. Furthermore, at least 9/57 (15.8%) of captive lion skulls showed FM stenosis (FMH <14.06 mm, the maximal FMH measured in six confirmed cases of FM stenosis with related neurological illness), suggesting that they may have suffered from, or at least were predisposed to, neurologic abnormality. In contrast, FM stenosis (FMH <14.06 mm) was detected in only two of the 473 skulls (0.4%) of wild lions studied. The cutoff value may be even higher, thus including a larger proportion of the skulls from captivity and only marginally more of those from the wild as was evident when the 5^th^ percentile of the FMH in the wild population was used as the cutoff value. Interestingly, no differences were found between the captive and wild tiger skulls in FMH or in the prevalence of skulls with an opening smaller than the 5^th^ percentile of the FMH in the wild tiger population. We therefore suggest that the pathology may be far less prevalent, if it exists at all, in wild lions and in captive and wild tigers. Unfortunately, little information was available to us concerning the histories of many of the skulls. However, it may be said that even if skulls of some wild-caught lions were included in the captive population, their influence was not strong enough to alter the fact that captive lions have smaller FMH.

Captivity is known to cause phenotypic changes as previously described by Darwin [27]. In captive lions, significant morphological differences were recorded in maxillary and mandibular regions [28] as well as in increased zygomatic arch breadth, shortening of the skull, reduced cranial volume, a general increase in the overall thickness of the skull and reduced FMH [29]. Malformation of the external occipital protuberances [30] and decrease in FMH [21] have been only rarely noticed in captive tigers. Captive felids are negatively affected by their inability to engage in normal hunting behaviors and these skull morphology changes were explained by different mechanical properties of food and altered muscle use in captivity during feeding, lack of exercise and the need to hunt, and by excessive grooming [29]–[32]. The skull and teeth appear to be the prime areas in which one can observe morphological responses to captive lifestyles [33]. These factors are for the most part non-hereditary, affect the individual animal, and include behavioral, morphological, and physiological changes. Howell [18], who re-examined Hollister's [29] specimens, suggested dietary deficiency as the cause of the observed differences. Wild lions and tigers hunt whole animals and eat the carcass with the internal organs. They are therefore able to selectively choose internal organs, possibly based on their nutritional content. In contrast, captive carnivores typically receive cut pieces of red meat, chicken or processed cat food rather than an entire carcass. This practice has been reported to cause dietary deficiencies in captive lions [11], [31].

Studies on nutritional deficiencies in captive lions and cheetahs showed a possible connection between vitamin deficiencies and the red meat diet fed to them [8], [11], [29], [34]. In growing dogs, a diet deficient in vitamin A has been shown to cause bone proliferation, resulting in thickened and enlarged bones of the skull, similar to what is found in lions that died from the neurological disease associated with FM stenosis [35]. Our findings demonstrated significant shortening of the FMH in captive lions. High variability in FMH was found in lions in general and more so in captive specimens. Furthermore, lion skulls with smaller FMH in both sexes were wider and had smaller volume when compared to skulls with normal opening. These are consistent with the possible correlation between malformation of bones of the skull, and specifically those surrounding the caudal fossa, and vitamin A availability. Levels of vitamin A in livers of captive lions that died due to this bone pathology were found to be very low, thus providing further support to the hypothesis that vitamin A deficiency is the primary cause of the investigated malformation [8], [11]. A more recent study demonstrated low levels of vitamin A in the liver of neurologically healthy living lions in captivity [36], suggesting that higher levels of vitamin A may be needed by young lions only at a certain phase of development. However, other large carnivores such as the cheetah or tiger only rarely demonstrate such malformations, as evident by just two reports available in the scientific literature [20], [21]. This seems consistent with the absence of significant difference in skull measurements between captive and wild tigers seen in this study. Such species differences may imply that zoo or wild status alone cannot explain the morphological changes in lions. In a study on vitamins A and E in various species in captivity [37], it was shown that while lions and tigers had the same vitamin A intake (38.2 mg/d), mean plasma all-trans-retinol level was 0.23 mg/L (range: 0.17–0.36) in tigers while it was only 0.17 mg/L (range: 0.10–0.21) in lions. This difference in plasma retinol level between lions and tigers may suggest a difference in dietary vitamin A absorbance efficiency between these two species. This may thus suggest possible species-specific genetic predisposition of the lion to morphological changes involving the area of the FM, changes that may be associated with a potential vitamin A deficiency.

Captive lions skulls showed greater variance in FMH, when compared to the wild population, suggesting existence of a captivity-related specific factor that alters the shape and size of bones surrounding the FM. Comparison of lions with FM stenosis (FMH equal to or smaller than 16.60 mm) to those with FMH larger than the 5^th^ percentile cutoff value indicated that skulls with FM stenosis were wider and had smaller cranial volume. We hypothesize here that these differences arise from additional bone formation that increases the total size of the skull while decreasing the space available for the brain. In other words, it may be proposed that additional bone growth is not only outward but also inward, possibly causing diminution of the caudal fossa, compression of the hindbrain and the neurological symptoms often seen in diseased lions.

Morphological differences between captive and wild populations can be attributed to both the power of selection, which is hereditary, produces genotypic changes, and affects the population as a whole, and environmental factors that may affect the individual [31]. A possible genetic basis for the malformation has not yet been investigated, although in some reports affected lions came from the same litter ([10], [17], [38]; Shamir et al. unpublished data), thus suggesting possible genetic, *in utero*, or postpartum factors. This may be related to a possible small founder population and subsequent inbreeding of lions in captivity. Yet, despite its possible genetic origins, this malformation has not been purged out of the captive population. We can only hypothesize on the reasons for that. One possible explanation is that a few carriers of the genetic alteration do reach adulthood and reproduce. Alternatively, the malformation persists in the captive population because it is caused by a combination of genetic predisposition and environmental factors such as nutrition.

## Conclusions

Using museum collections enabled us to examine a large number of skulls. However, important information such as specific habitat description, exact age, available diet, medical status, and cause of death was often not available. Our findings suggest a possible predisposition for abnormal bone growth and decreased FMH in skulls of captive lions in comparison to those of wild lions (within-species comparison) as well as to the tiger, another obligatory carnivore of similar size (across-species comparison). Whether these abnormalities are related to insufficient nutrients such as vitamin A or to other environmental factors in captivity and/or to genetic factors has yet to be determined. Detection of the same bone malformation in captive lions for more than 500 years highlights a need for further investigation with a view to reducing its occurrence.
